# Sex-specific trunk movement coordination in participants with low-back pain and asymptomatic controls

**DOI:** 10.3389/fspor.2025.1524489

**Published:** 2025-04-01

**Authors:** Lukas Fischer, Arno Schroll, Hendrik Schmidt, Adamantios Arampatzis

**Affiliations:** ^1^ Department of Training and Movement Sciences, Humboldt-Universität zu Berlin, Berlin, Germany; ^2^ Berlin School of Movement Science, Humboldt-Universität zu Berlin, Berlin, Germany; ^3^ Julius Wolff Institute, Berlin Institute of Health at Charité—Universitätsmedizin Berlin, Berlin, Germany

**Keywords:** lumbo-pelvic rhythm, spine alignment, trunk stability, trunk variability, lordosis

## Abstract

**Background:**

Trunk posture and lumbo-pelvic coordination can influence spinal loading and are commonly used as clinical measures in the diagnosis and management of low-back pain and injury risk. However, sex and pain specific characteristics have rarely been investigated in a large cohort of both healthy individuals and low-back pain patients. It has also been suggested that the motor control of trunk stability and trunk movement variability is altered in individuals with low-back pain, with possible implications for pain progression. Nonetheless, clear links to low-back pain are currently lacking.

**Objective:**

To investigate trunk posture, lumbo-pelvic coordination, trunk dynamic stability and trunk movement variability in an adequately large cohort of individuals with low-back pain and asymptomatic controls and to explore specific effects of sex, pain intensity and pain chronicity.

**Methods:**

We measured lumbo-pelvic kinematics during trunk flexion and trunk dynamic stability and movement variability during a cyclic pointing task in 306 adults (156 females) aged between 18 and 64 years, reporting either no low-back pain or pain in the lumbar area of the trunk. Participants were grouped based on their characteristic pain intensity as asymptomatic (ASY, *N* = 53), low to medium pain (LMP, *N* = 185) or medium to high pain (MHP, *N* = 68). Participants with low-back pain that persisted for 12 weeks or longer were categorized as chronic (*N* = 104). Data were analyzed using linear mixed models in the style of a two way anova.

**Results:**

Female participants showed a higher range of motion in both the trunk and pelvis during trunk flexion, as well as an increased lumbar lordosis in standing attributed to a higher pelvic angle that persisted throughout the entire trunk flexion movement, resulting in a longer duration of lumbar lordosis. The intensity and chronicity of the pain had a negligible effect on trunk posture and the lumbo-pelvic coordination. Pain chronicity had an effect on trunk dynamic stability (i.e., increased trunk instability), while no effects of sex and pain intensity were detected in trunk dynamic stability and movement variability.

**Conclusions:**

Low-back pain intensity and chronicity was not associated with lumbo-pelvic posture and kinematics, indicating that lumbo-pelvic posture and kinematics during a trunk flexion movement have limited practicality in the clinical diagnosis and management of low-back pain. On the other hand, the increased local instability of the trunk during the cyclic coordination task studied indicates control errors in the regulation of trunk movement in participants with chronic low-back pain and could be considered a useful diagnostic tool in chronic low-back pain.

## 1 Introduction

Low-back pain (LBP) is a leading cause of years lived with disability for 568 million people world wide (1) and is the leading health condition contributing to the need for rehabilitation services globally (2), with immense health care costs and loss of productivity regardless of the treatment indicating the need for nuanced interventions (3). Range of motion (RoM), posture and alignment of the spine during trunk flexion have often been used as key components in the clinical diagnosis of people with low-back pain (4, 5). Trunk flexion in the sagittal plane is a frequently performed movement especially in work-related activities (6) and earlier studies have associated trunk flexion movements with an increased load on the spine by measuring intradiscal pressures *in vivo* (7–9) and using musculoskeletal models (10–12). Trunk flexion involves both lumbar and pelvic tilt, and quantifying lumbo-pelvic coordination has potential applications in load and injury-risk assessment (13–16) and low-back pain therapy (17, 18). Although the lumbo-pelvic coordination during trunk flexion and extension movements has been extensively studied (19–22), determinants such as sex- or pain-specific characteristics have rarely been examined. Some studies (19) found no differences in lumbo-pelvic coordination between female and male participants with and without low-back pain, whereas others (23) reported sex-specific differences in lumbo-pelvic coordination in asymptomatic participants. A recent systematic review (24) found an effect of sex on lumbar lordosis and lumbar RoM, but noted that the studies available for synthesis were limited and more evidence was needed. Considering the importance of lumbar lordosis in spinal loading (11, 16, 25) and its possible association with low-back pain and spondylolysis (26, 27) an assessment of lumbo-pelvic kinematics in a large number of healthy participants and patients with low-back pain may be of clinical relevance.

It is generally accepted that the risk of developing low-back pain is multifactorial (28–31) and that besides psychosocial (32–34) and personal factors (35–37), a deterioration of the motor control of spinal stability may be related to the onset and progression of low-back pain (13, 38–40). Non-linear analyses can be used to characterize the motor control of trunk stability during repetitive dynamic trunk movements (41). The local dynamic stability of the trunk can be assessed from kinematic data using the maximum finite-time Lyapunov exponent (41). Positive values of this exponent show a divergence of the nearest neighbors in state space over time, while larger values reveal a faster divergence (42), thus indicating a greater effect of small perturbations in trunk kinematics. While there are indications of an association between pathological conditions and trunk movement variability (43, 44) it should be noted that movement variability may also be of functional relevance and indicate a skilled repertoire of a healthy motor system (45) that is needed to cope with perturbations (46). Investigations that incorporate the temporal structure of the movement, such as non-linear time series analyses, can provide insight into the motor control of a systems stability (39, 47) and help to understand the effect of low-back pain on movement (48). However, the effect of low-back pain on trunk dynamic stability and trunk movement variability is currently not well understood (49). Previous studies found no clear effect of low-back pain on trunk dynamic stability (50) and movement variability (51). Current reviews find insufficient evidence (51) and report inconsistent results (52) probably due to differences in methods, task demands and inclusion criteria. It has been suggested that subgroups according to pain characteristics (52, 53), analyses of possible confounders and a large number of participants included (51) may help to detect low-back specific adjustments in spinal motion, thereby increasing the clinical utility of certain measurement variables.

The purpose of the current study was to investigate trunk posture, lumbo-pelvic coordination, trunk dynamic stability and trunk movement variability in an adequately large cohort of participants with low-back pain and asymptomatic controls and to explore specific effects of sex, pain intensity and pain chronicity. Based on literature reports of sex-specific characteristics in lumbar lordosis, sacrum orientation and lumbo-pelvic rhythm (23, 54) we hypothesized that females would have higher lumbar lordosis, higher pelvic RoM and lower lumbo-pelvic ratio than males. Furthermore, based on the reported inconsistent findings of low-back pain on spinal posture and movement (55), we hypothesized that pain intensity would not affect the investigated variables, but that chronic low-back pain would, due to possible control errors in the regulation of trunk movement.

## 2 Methods

### 2.1 Experimental design

To determine an appropriate sample size for six groups (i.e., three levels of pain intensity, two levels of sex), we conducted an *a priori* power analysis (G*Power 3.1.9.7) using the outcomes in measures of lumbo-pelvic coordination from an earlier study by our group (56), where we observed medium effect sizes from 0.5 to 0.7 (Cohen's *d*) between male and female participants. Assuming a more conservative medium effect size of *f* = 0.20, an alpha error of 0.05 and a statistical power of 0.8 for a balanced group design, a total of 244 participants should suffice to detect specific differences between pain groups or interaction effects (*df* = 2) in measures of lumbo-pelvic coordination. To detect differences between male and female participants (*df* = 1), a total of 199 participants would be sufficient. Based on this power analysis and an assumed data loss ratio of at least 20% due to drop out or data quality, we recruited 306 adults for this study (156 females, 150 males, Table 1). We included participants aged between 18 and 64 years, reporting either no low-back pain or pain in the lumbar area of the trunk. Exclusion criteria were as follows: body mass index (BMI) >28, central or peripheral neurological impairments, prior spine surgery, malposition or aberration of lower extremities, pregnancy, medication with opioids or muscle relaxants, rheumatism, osteoporosis or acute infection, cardiac diseases. Study participants were recruited within the ongoing “Berlin Back Study”—a prospective cross-sectional investigation registered with the German Clinical Trial Register (DRKS-ID: DRKS00027907, DRKS00029361). The recruitment of participants was conducted through multiple channels, including local promotion at Charité-Universitätsmedizin Berlin (via mailed flyers, notice boards, online platforms, and social media), public outreach (including newspapers, magazines, podcasts, and television), collaborations with local businesses and administrative bodies, and word-of-mouth referrals. The study protocol adheres to the ethical principles outlined in the Helsinki Declaration (57). The study follows the STROBE guidelines (58) and was approved by the Ethics Committee of the Humboldt-Universität zu Berlin (HU-KSBF-EK_2021_0006). Written informed consent was obtained from all participants.

**Table 1:** Participants' anthropometric characteristics.

	ASY		LMP		MHP		*p*-values			
	Male	Female	Male	Female	Male	Female	cLBP	Sex	Pain	Int
	(*N* = 26)	(*N* = 27)	(*N* = 91)	(*N* = 94)	(*N* = 33)	(*N* = 35)				
Age [y]	36.50 ± 10.08	38.15 ± 14.75	42.60 ± 11.02	42.02 ± 12.63	41.93 ± 14.13	39.69 ± 12.30	0.582	0.797	0.103	0.513
Height [m]*	1.80 ± 0.08	1.66 ± 0.07	1.80 ± 0.08	1.67 ± 0.06	1.81 ± 0.14	1.69 ± 0.07	0.350	**<0** **.** **001**	0.698	0.662
Mass [kg]*^,‡,§^	78.13 ± 11.35	62.66 ± 7.67	79.39 ± 9.79	63.48 ± 9.32	80.51 ± 12.12	68.49 ± 10.48	0.525	**<0** **.** **001**	**0** **.** **040**	0.264
BMI [kg/m^2^]*^,‡,§^	24.07 ± 2.85	22.71 ± 2.57	24.47 ± 2.72	22.70 ± 2.97	24.90 ± 4.26	23.89 ± 3.32	0.144	**<0** **.** **001**	**0** **.** **022**	0.531
CPI^†^^,^^‡^^,^^§^	0.00 ± 0.00	0.00 ± 0.00	25.31 ± 12.63	26.21 ± 12.10	60.30 ± 8.71	61.62 ± 10.27	**<0** **.** **001**	0.627	**<0** **.** **001**	0.955

* Significant differences (*p* < 0.05) between male and female.

^†^ Significant differences (*p* < 0.05) between ASY and LMP, *post-hoc* analysis.

^‡^ Significant differences (*p* < 0.05) between ASY and MHP, *post-hoc* analysis.

^§^ Significant differences (*p* < 0.05) between LMP and MHP, *post-hoc* analysis.

Values are presented as mean ± standard deviation.

ASY: asymptomatic no pain (CPI = 0); LMP: low to medium pain (CPI = 1–49); MHP: medium to high pain (CPI = 50–100).

*p*-values denote effects of pain chronicity (cLBP), sex (Sex), pain intensity (Pain) or a sex-by-pain interaction (Int).

Bold values highlight significant differences (*p* < 0.05).

### 2.2 Pain assessment

Pain intensity was assessed using the validated German version (59) of the Graded Chronic Pain Scale (60). The characteristic pain intensity (CPI) (61) was calculated as the average of 0–10 ratings of pain right now, average pain and worst pain in the last three months multiplied by 10 resulting in a score between 0 and 100. CPI scores were then used to classify all participants as either (1) asymptomatic (ASY, CPI = 0) (2), participants with low to medium pain (LMP, CPI = 1–49), or (3) participants with medium to high pain (MHP, CPI = 50–100) (60). The chronicity of low-back pain was assessed for all participants during the clinical examination conducted by an orthopedic and trauma surgery specialist. Low-back pain that persisted for 12 weeks or longer was defined as chronic (62).

### 2.3 Lumbo-pelvic kinematics

**Figure 1:**
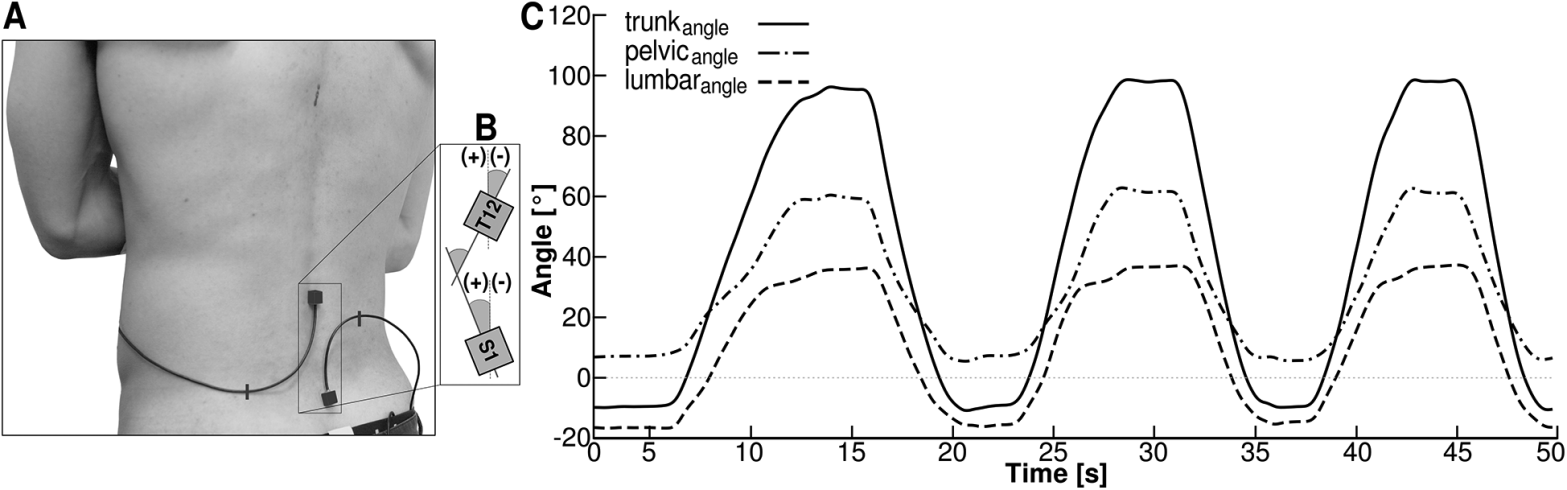
**(A)** Two 3-dimensional accelerometers positioned at sacral vertebrae 1 (S1) and thoracic vertebrae 12 (T12) were used to measure the pelvic (pelvic_angle_) and trunk (trunk_angle_) angles during three trunk flexion movements. **(B)** Positive values (+) indicate a forward-rotated orientation with respect to the vertical, while negative values (−) indicate a backward-rotated orientation of the pelvis or trunk. The lumbar angle (lumbar_angle_) was calculated as the difference between trunk_angle_ and pelvic_angle_. **(C)** Angles of trunk, pelvis and lumbar spine during the three trunk flexion movements. The range of motion of the pelvis (pelvic_RoM_), the trunk (trunk_RoM_) and the lumbar spine (lumbar_RoM_) was calculated as the difference of the respective maximum angle during the movement and the respective angle in the upright standing position. Values of the lumbar angle less than zero indicate the phase of lumbar lordosis while values of the lumbar angle above zero indicate the phase of lumbar kyphosis.

All participants performed three consecutive trunk forward bending movements. They were instructed to perform the movement in a slow but comfortable pace without bending their knees and until the individual maximal trunk flexion position. The lumbo-pelvic alignment and range of motion of both the trunk and pelvis were measured using two 3-dimensional accelerometers (Biovision, Berlin; size 1 × 1 × 1 cm, 500 Hz). The sensors were attached to the skin at the level of thoracic vertebrae 12 (T12) and sacral vertebrae 1 (S1) using double-sided adhesive tape (Figure 1A).

The obtained acceleration data were low pass filtered at 5 Hz using a 2nd order IIR Butterworth zero-phase filter and processed using a moving average in a time window of 500 ms. The orientation of the local coordinate system of each sensor in reference to the global space coordinate system was determined using the gravitational field. To minimize the influence of accelerations additional to the gravitational acceleration, participants were encouraged to execute the movement at a low, controlled velocity and only trials in which the norm of the gravitational vector was 1 ± 0.10 g were included in further calculations. During the trunk forward bending movement the angles of the pelvis and the trunk were measured in the sagittal plane using the orientation of the local coordinate systems of the sensors at S1 and T12 with respect to the global coordinate system. Positive values of the two angles indicate a forward-rotated orientation (with respect to the vertical), while negative values indicate a backward-rotated orientation of the pelvis or the trunk (Figure 1B). The difference between the trunk angle and the pelvic angle was used to calculate the lumbar angle with negative values indicating the magnitude of lumbar lordosis. Finally, a moving average filter was applied to the calculated angle values.

To identify start and end points of each cycle, we calculated secant slopes of the trunk angle in time increments of 100 ms. The start of a cycle was determined by the secant slope value falling below 0.1 and the end of a cycle was defined by the maximum angle value. Each cycle was time normalized to 1,000 data points and from the three performed cycles a mean trial was calculated. The range of motion of the pelvis (pelvic_RoM_), the trunk (trunk_RoM_) and the lumbar spine (lumbar_RoM_) was calculated as the difference of the respective maximum angle value during the trunk forward bending movement and the respective angle value in the upright standing position (Figure 1C). During the forward bending movement the duration of the lumbar lordosis and the duration of the lumbar lordosis normalized to the movement time was quantified from movement start to the first occurrence of a positive value in the lumbar angle. Further, we quantified the changes of the pelvic angle during the lumbar lordosis (pelvic_RoM−Lordosis_), during the lumbar kyphosis (pelvic_RoM−Kyphosis_) and the pelvic angle at the end of the lordotic phase to identify the orientation of the pelvis during the transition from lordosis to kyphosis. Finally, the lumbo-pelvic ratio (LPR) was calculated as the ratio of the changes in lumbar spine orientation to the changes in pelvic orientation for the whole movement (LPR_full_) and for the lumbar lordosis (LPR_Lordosis_) and kyphosis (LPR_Kyphosis_) phases of the movement.

### 2.4 Trunk dynamic stability

**Figure 2:**
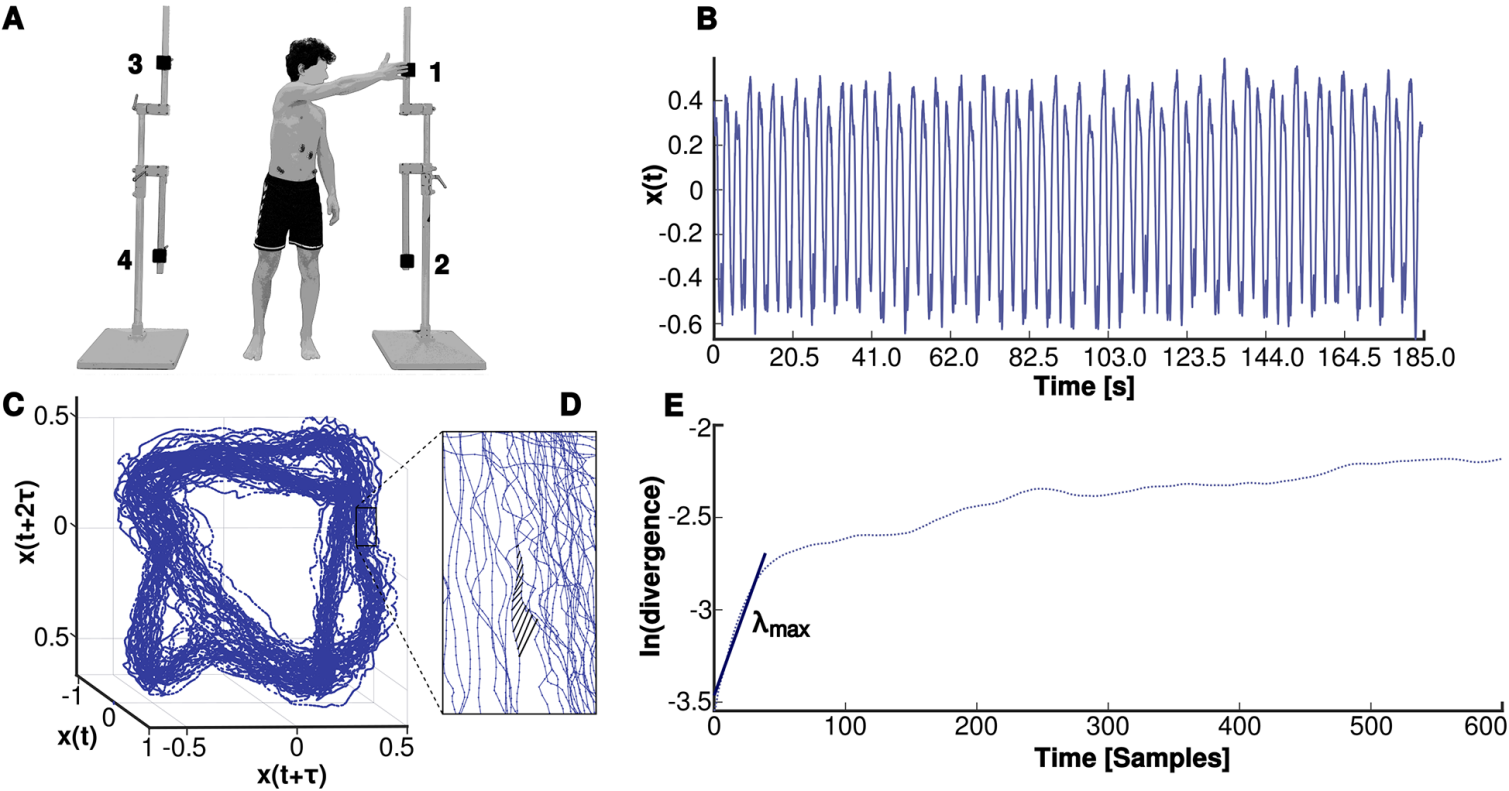
Movement task and assessment of the local dynamic stability of the trunk. **(A)** Participants repeatedly executed a pointing task from position 1 over 2 with the right arm and 3–4 with the left arm with a frequency of 10 cycles per minute (0.17 Hz); **(B)** one-dimensional time-series *x*(*t*) derived from the norm of the 3-dimensional accelerations of the trunk (accelerometer at the level of the second thoracic vertebrae); **(C)** reconstructed state space of the trunk movement using dimension *m* = 3 and time delay *τ* = 75; **(D)** diverging Euclidean distances of a nearest neighbor pair in the reconstructed state space; **(E)** average logarithmic rate (ln) of divergence of all nearest neighbor pairs over time and the maximum Lyapunov exponent (*λ*_max_) as slope of the linear fit to the resulting ln(divergence) curve for 0–40 samples.

The participants performed a cyclic movement that comprised trunk rotation and flexion by reaching targets positioned on the left and right side of the participant (Figure 2A). The targets were mounted on metal frames and adjusted to match the participants height at eye level and the proximal border of the patella. The frames were adjusted in width to the arm span width of the respective participant. The movement was explained and demonstrated to the participants and the movement frequency of 10 cycles per minute (0.17 Hz) was introduced by a digital metronome. After a short familiarization phase participants continuously performed a total of 32 movement cycles.

The local trunk dynamic stability was examined using the maximum finite-time Lyapunov exponent (*λ*_max_). Kinematic data were collected using a 3-dimensional accelerometer (Biovision, Berlin; size 1 × 1 × 1 cm, 500 Hz) attached to the skin at the level of the second thoracic vertebrae (T2). The obtained acceleration data were low pass filtered at 20 Hz using a 2nd order IIR Butterworth zero-phase filter. We calculated the norm of the measured 3-dimensional accelerations after subtracting the respective minima. The resulting one-dimensional time-series was demeaned and a total of 30 cycles were used for subsequent analysis (Figure 2B). The time-series was time normalized to 18,000 data points (600 data points per cycle) and the reconstruction of the trunk motion in state space (Figures 2C,D) was performed using the method of delay embedding by choosing an appropriate time delay *τ* and embedding dimension *m* as follows (Equation 1):

(1) S(t)=[x(t),x(t+τ),x(t+2τ),…,x(t+(m−1)τ)]

with *S*(*t*) representing the *m*-dimensional reconstructed state vector, *x*(*t*) the one-dimensional Euclidean norm series, *τ* the time delay, and *m* the embedding dimension. For each time series a constant time delay of *τ* = 75 was appropriate based on average mutual information analysis (63). Global false nearest neighbor's analysis (64) revealed dimension *m* = 3 to be sufficient for the reconstruction of the current data. Finally, the maximum Lyapunov exponent was calculated as the slope of the logarithmic average divergence curve using the algorithm of Kantz (65) for the first 40 values (Figure 2E). This parameter describes the average logarithmic divergence between initially neighboring trajectories in state space. Thus, the higher the maximum Lyapunov exponent, the more unstable the system responds locally to external mechanical induced perturbations (66) or to internal motor control perturbations (67). To quantify the variability of the trunk movement, the time series of the norm of the measured 3-dimensional accelerations during all 30 cycles was used. Each cycle was time normalized to 101 data points (0%–100%) and standard deviations were calculated across all cycles at every time point. Finally, the mean variability (*MeanSD*) was calculated over all values for each trial (68).

## 3 Statistics

To account for possible effects of sex, pain intensity and pain chronicity on lumbo-pelvic coordination, trunk dynamic stability and trunk movement variability, we analyzed the data using linear mixed models in the style of a two way anova with interaction term with the factors sex and pain intensity and the covariate pain chronicity for the anthropometric characteristics (i.e., age, height, mass, BMI) and the kinematic parameters (i.e., movement time, trunk_RoM_, pelvic_RoM_, lumbar_RoM_, lumbar angle at beginning and end of trunk flexion, pelvic angle at beginning and end of trunk flexion and lumbar lordosis, pelvic_RoM−Lordosis_, pelvic_RoM−Kyphosis_, duration of lumbar lordosis, normalized duration of lumbar lordosis, LPR_full_, LPR_Lordosis_, LPR_Kyphosis_, variability, maximum Lyapunov Exponent) by using the generalized least squares (gls) approach (69) implemented in the package “nlme” (70). In the case of a significant main or interaction effect we performed a *post-hoc* analysis with controlling the false discovery rate using the approach by Benjamini and Hochberg (71). All the significance levels were set to *α* = 0.05 and analyses were conducted in R (72).

## 4 Results

Participants showed no significant differences in age (*p* = 0.103) or body height (*p* = 0.698) between the different pain groups (Table 1). However, male participants were significantly taller (*p* < 0.001), heavier (*p* < 0.001) and had a higher BMI (*p* < 0.001) than females. In the body mass (*p* = 0.040) and BMI (*p* = 0.022) of the participants we identified a significant main effect of pain intensity. Participants with medium to high pain had a higher BMI than asymptomatic participants (*p* = 0.027) and participants with low to medium pain (*p* = 0.027). No effect of pain chronicity was detected in age (*p* = 0.582), body height (*p* = 0.350), body mass (*p* = 0.525), and BMI (*p* = 0.144) of the participants.

In the time of the forward trunk bending movement no effect of sex (*p* = 0.132), pain intensity (*p* = 0.052), pain chronicity (*p* = 0.274) or a sex-by-pain interaction (*p* = 0.105) was detected (Table 2). In the trunk_RoM_ we identified a significant main effect of sex (*p* < 0.001) and pain intensity (*p* = 0.009) but no effect of pain chronicity (*p* = 0.440) or a sex-by-pain interaction (*p* = 0.479). Females demonstrated a higher trunk_RoM_ compared to males (*p* < 0.001). In participants with medium to high pain trunk_RoM_ was significantly reduced when compared to asymptomatic participants (*p* = 0.006). We did not detect statistically significant differences in trunk_RoM_ between participants with low to medium pain and participants with medium to high pain (*p* = 0.087). In the pelvic_RoM_ we detected a significant main effect of sex (*p* < 0.001) but no effect of pain intensity (*p* = 0.069), pain chronicity (*p* = 0.998) or a sex-by-pain interaction (*p* = 0.223). Females demonstrated a higher pelvic_RoM_ compared to males (*p* < 0.001). In the lumbar_RoM_ we did not find any significant main effect of sex (*p* = 0.594), pain intensity (*p* = 0.052), pain chronicity (*p* = 0.346) or a sex-by-pain interaction (*p* = 0.675). The lumbar angle in the upright standing position demonstrated a significant main effect of sex (*p* < 0.001) and pain intensity (*p* = 0.002), but no effect of pain chronicity (*p* = 0.921) or a sex-by-pain interaction (*p* = 0.437; Table 3). Female participants had a significantly greater lumbar angle in the upright standing position (i.e., higher lumbar lordosis; *p* < 0.001). Participants with low to medium pain showed a significantly reduced lumbar angle in the upright standing position (i.e., smaller lumbar lordosis) when compared to asymptomatic participants (*p* = 0.049) and participants with medium to high pain (*p* = 0.049). We did not detect statistically significant differences in the lumbar angle during upright standing between participants with medium to high pain and asymptomatic participants (*p* = 0.929). In the lumbar angle at the end of the movement we detected a significant main effect of sex (*p* < 0.001) but no effect of pain intensity (*p* = 0.203), pain chronicity (*p* = 0.217) or a or a sex-by-pain interaction (*p* = 0.654). Females showed a significantly smaller lumbar angle at the end of the movement (i.e., less kyphosis) than males (*p* < 0.001).

**Table 2:** Movement time (time_mov_), range of motion of the trunk (trunk_RoM_), pelvis (pelvic_RoM_) and lumbar spine (lumbar_RoM_) of participants with different pain levels.

	ASY		LMP		MHP		*p*-values			
	Male	Female	Male	Female	Male	Female	cLBP	Sex	Pain	Int
	(*N* = 26)	(*N* = 27)	(*N* = 91)	(*N* = 94)	(*N* = 33)	(*N* = 35)				
time_mov_ [s]	8.38 ± 1.67	7.95 ± 2.43	8.41 ± 2.96	9.28 ± 3.01	8.04 ± 2.56	8.09 ± 2.78	0.274	0.132	0.052	0.105
trunk_RoM_ [°]*^,‡^	110.81 ± 12.45	117.94 ± 13.93	103.71 ± 17.09	114.59 ± 14.61	101.15 ± 17.47	109.45 ± 17.32	0.440	**<0** **.** **001**	**0** **.** **009**	0.479
pelvic_RoM_ [°]*	68.79 ± 13.79	75.74 ± 16.92	64.90 ± 16.19	76.80 ± 16.82	64.07 ± 16.91	68.48 ± 13.77	0.998	**<0** **.** **001**	0.068	0.223
lumbar_RoM_ [°]	42.03 ± 12.01	42.20 ± 12.36	38.81 ± 12.80	37.79 ± 13.22	37.08 ± 11.33	40.97 ± 12.75	0.346	0.594	0.052	0.675

* Significant differences (*p* < 0.05) between male and female.

^‡^ Significant differences (*p* < 0.05) between ASY and MHP, *post-hoc* analysis.

Values are presented as mean ± standard deviation.

ASY: asymptomatic no pain (CPI = 0); LMP: low to medium pain (CPI = 1–49); MHP: medium to high pain (CPI = 50–100).

*p*-values denote effects of pain chronicity (cLBP), sex (Sex), pain intensity (Pain) or a sex-by-pain interaction (Int).

Bold values highlight significant differences (*p* < 0.05).

**Table 3:** Angle of the lumbar spine in the upright standing position and at the end of the movement, pelvic angle during standing, at the end of the lumbar lordosis and at the end of the movement, trunk angle during standing and at the end of the movement, changes in pelvic angle during the phases of lumbar lordosis (pelvic_ROM−lordosis_) and lumbar kyphosis (pelvic_ROM−kyphosis_), mean duration of the lumbar lordosis (LOR_dur_), mean duration of the lumbar lordosis relative to the movement time (LOR_dur−norm_) for participants with different pain levels.

	ASY		LMP		MHP		*p*-values			
	Male	Female	Male	Female	Male	Female	cLBP	Sex	Pain	Int
	(*N* = 26)	(*N* = 27)	(*N* = 91)	(*N* = 94)	(*N* = 33)	(*N* = 35)				
lumbar angle stand [°]*^,†,§^	−20.30 ± 10.26	−29.38 ± 16.14	−17.63 ± 10.54	−23.84 ± 13.78	−19.38 ± 9.91	−29.91 ± 12.30	0.921	**<0** **.** **001**	**0** **.** **002**	0.437
lumbar angle end [°]*	21.72 ± 8.76	12.82 ± 9.39	21.18 ± 11.33	13.95 ± 10.07	17.71 ± 11.34	11.06 ± 10.42	0.217	**<0** **.** **001**	0.203	0.654
pelvic angle stand [°]*^,†^	7.89 ± 8.07	15.66 ± 13.58	6.30 ± 9.94	11.97 ± 11.66	7.52 ± 8.63	17.47 ± 9.45	0.157	**<0** **.** **001**	**0** **.** **008**	0.364
pelvic angle lordosis [°]*	36.61 ± 19.53	59.85 ± 27.63	33.96 ± 21.32	52.13 ± 27.52	38.01 ± 19.09	56.43 ± 25.06	0.931	**<0** **.** **001**	0.201	0.945
pelvic angle end [°]*^,‡^	76.68 ± 12.64	91.40 ± 13.78	71.20 ± 15.47	88.77 ± 14.85	71.59 ± 13.72	85.95 ± 16.07	0.340	**<0** **.** **001**	**0** **.** **036**	0.654
trunk angle stand [°]	−12.42 ± 4.57	−13.72 ± 6.85	−11.34 ± 6.49	−11.87 ± 6.58	−11.85 ± 4.79	−12.44 ± 7.66	**0** **.** **042**	0.292	0.252	0.888
trunk angle end [°]*^,‡^	98.40 ± 11.10	104.22 ± 12.59	92.38 ± 16.39	102.72 ± 14.68	89.30 ± 18.04	97.01 ± 15.31	0.947	**<0** **.** **001**	**0** **.** **003**	0.400
pelvic_RoM−Lordosis_ [°]*	28.72 ± 15.45	41.49 ± 24.28	27.00 ± 16.22	38.76 ± 25.14	29.40 ± 16.76	38.55 ± 20.00	0.399	**<0** **.** **001**	0.583	0.840
pelvic_RoM−Kyphosis_ [°]^§^	40.07 ± 18.58	37.32 ± 29.76	38.80 ± 21.79	39.69 ± 27.57	36.45 ± 22.69	31.04 ± 20.88	0.322	0.391	**0** **.** **027**	0.733
LOR_dur_ [s]*	2.95 ± 1.16	3.88 ± 2.57	3.05 ± 1.85	4.40 ± 3.01	3.11 ± 1.79	4.09 ± 2.23	0.234	**<0** **.** **001**	0.299	0.616
LOR_dur−norm_ [%]*	35.78 ± 12.92	52.45 ± 21.09	38.89 ± 19.64	48.21 ± 23.96	43.34 ± 21.38	52.26 ± 22.25	0.837	**<0** **.** **001**	0.103	0.590

* Significant differences (*p* < 0.05) between male and female.

^†^ Significant differences (*p* < 0.05) between ASY and LMP, *post-hoc* analysis.

^‡^ Significant differences (*p* < 0.05) between ASY and MHP, *post-hoc* analysis.

§ Significant differences (*p* < 0.05) between LMP and MHP, *post-hoc* analysis.

Values are presented as mean ± standard deviation.

ASY: asymptomatic no pain (CPI = 0); LMP: low to medium pain (CPI = 1–49); MHP: medium to high pain (CPI = 50–100).

*p*-values denote effects of pain chronicity (cLBP), sex (Sex), pain intensity (Pain) or a sex-by-pain interaction (Int).

Bold values highlight significant differences (*p* < 0.05).

In the pelvic angle during the upright standing position we identified a significant main effect of sex (*p* < 0.001) and pain intensity (*p* = 0.008), but no effect of pain chronicity (*p* = 0.157) or a sex-by-pain interaction (*p* = 0.364). Female participants had a significantly higher pelvic angle in the upright standing position (*p* < 0.001). In female participants with low to medium pain the pelvic angle in the upright standing position was significantly smaller than in asymptomatic female participants (*p* = 0.013) and female participants with medium to high pain (*p* = 0.047). In the pelvic angle at the end of the lordotic phase we identified a significant main effect of sex (*p* < 0.001) but no effect of pain intensity (*p* = 0.201), pain chronicity (*p* = 0.931) or a sex-by-pain interaction (*p* = 0.945). Female participants showed significantly higher pelvic angles at the end of the lordotic phase than male participants (*p* < 0.001). In the pelvic angle at the end of the movement we identified a significant main effect of sex (*p* < 0.001) and pain intensity (*p* = 0.036), but no effect of pain chronicity (*p* = 0.340) or a sex-by-pain interaction (*p* = 0.654). Female participants showed significantly higher pelvic angles at the end of the movement than male participants (*p* < 0.001). Participants with medium to high pain showed a significantly reduced pelvic angles at the end of the movement when compared to asymptomatic participants (*p* = 0.031).

In the trunk angle during the upright standing position we identified a significant effect of pain chronicity (*p* = 0.042), but no effect of sex (*p* = 0.292), pain intensity (*p* = 0.252) or a sex-by-pain interaction (*p* = 0.888). Participants with chronic pain had a significantly lower trunk angle in the upright standing position (*p* = 0.042). In the trunk angle at the end of the movement we identified a significant main effect of sex (*p* < 0.001) and pain intensity (*p* = 0.003), but no effect of pain chronicity (*p* = 0.947) or a sex-by-pain interaction (*p* = 0.400). Female participants showed significantly higher trunk angles at the end of the movement than male participants (*p* < 0.001). Participants with medium to high pain showed a significantly reduced trunk angle at the end of the movement when compared to asymptomatic participants (*p* = 0.011).

In pelvic_RoM_ during the phase of lumbar lordosis we found a significant main effect of sex (*p* < 0.001), but no effect of pain intensity (*p* = 0.068), pain chronicity (*p* = 0.339) or a sex-by-pain interaction (*p* = 0.840). Females demonstrated significantly higher values in pelvic_RoM_ during the phase of lumbar lordosis than males (*p* < 0.001). In pelvic_RoM_ during the phase of lumbar kyphosis we detected a significant main effect of pain intensity (*p* = 0.027), but no effects of sex (*p* = 0.391), pain chronicity (*p* = 0.322) or a sex-by-pain interaction (*p* = 0.733). *post-hoc* analysis revealed no significant differences between the different pain groups. In the duration of lumbar lordosis and the duration of lumbar lordosis normalized to the movement time we detected a significant main effect of sex (*p* < 0.001), but no effect of pain intensity (*p* = 0.299 and *p* = 0.103), pain chronicity (*p* = 0.234 and *p* = 0.837) or a sex-by-pain interaction (*p* = 0.616 and *p* = 0.590). Females were significantly longer in a lordotic position when compared to males (*p* < 0.001).

**Figure 3:**
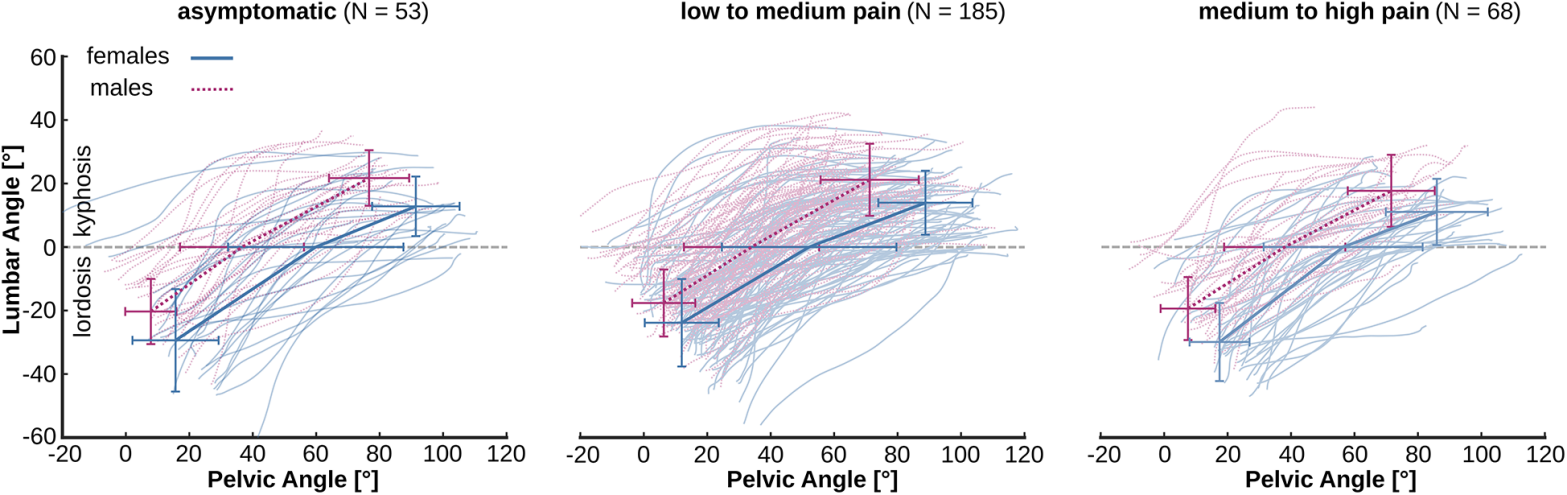
Individual lumbar angle as a function of pelvic angle for participants with different low-back pain intensities during trunk flexion. Crosses represent means ± standard deviations at upright standing, end of lumbar lordosis and end of movement, respectively. The average slope of each segment represents the corresponding lumbo-pelvic ratio.

In the LPR we detected a significant main effect of sex (*p* < 0.001) during the phase of the lumbar kyphosis, with females demonstrating a lower LPR during this phase of the movement (Table 4, Figure 3). No effect of pain intensity (*p* > 0.05), pain chronicity (*p* > 0.05) or a sex-by-pain interaction (*p* > 0.05) was evident in LPR_full_, LPR_Lordosis_ and LPR_Kyphosis_. Finally, in movement variability and the maximum Lyapunov Exponent during the cyclic pointing task no effect of sex (*p* = 0.822 and *p* = 0.543), pain intensity (*p* = 0.214 and *p* = 0.719) or a sex-by-pain interaction (*p* = 0.812 and *p* = 0.661) was detected. However, in the maximum Lyapunov Exponent we found a significant effect of pain chronicity (*p* = 0.008), with higher values in participants with chronic pain. In movement variability no effect of pain chronicity was detected (*p* = 0.247).

**Table 4:** Lumbo-pelvic ratio of the full movement (LPR_full_), during the phase of lumbar lordosis (LPR_Lordosis_) and lumbar kyphosis (LPR_Kyphosis_), variability of the trunk (VAR) and maximum lyapunov exponent (MLE) of the participants with different pain levels.

	ASY		LMP		MHP		*p*-values			
	Male	Female	Male	Female	Male	Female	cLBP	Sex	Pain	Int
	(*N* = 26)	(*N* = 27)	(*N* = 91)	(*N* = 94)	(*N* = 33)	(*N* = 35)				
LPR_full_	0.67 ± 0.35	0.61 ± 0.29	0.66 ± 0.35	0.54 ± 0.30	0.67 ± 0.51	0.63 ± 0.28	0.273	0.051	0.226	0.776
LPR_Lordosis_	0.84 ± 0.57	0.93 ± 0.47	0.85 ± 0.60	1.12 ± 1.52	1.11 ± 1.22	1.01 ± 0.64	0.109	0.410	0.939	0.440
LPR_Kyphosis_*	0.79 ± 0.71	0.46 ± 0.30	0.78 ± 0.71	0.48 ± 0.50	0.65 ± 0.45	0.50 ± 0.35	0.054	**<0** **.** **001**	0.945	0.676
VAR [m/s^2^]	0.38 ± 0.05	0.37 ± 0.06	0.38 ± 0.05	0.37 ± 0.06	0.36 ± 0.06	0.36 ± 0.07	0.247	0.822	0.214	0.812
MLE	2.44 ± 0.35	2.53 ± 0.42	2.54 ± 0.36	2.55 ± 0.38	2.52 ± 0.39	2.56 ± 0.31	**0** **.** **008**	0.543	0.719	0.661

* Significant differences (*p* < 0.05) between male and female.

Values are presented as mean ± standard deviation.

ASY: asymptomatic no pain (CPI = 0); LMP: low to medium pain (CPI = 1–49); MHP: medium to high pain (CPI = 50–100).

*p*-values denote effects of pain chronicity (cLBP), sex (Sex), pain intensity (Pain) or a sex-by-pain interaction (Int).

Bold values highlight significant differences (*p* < 0.05).

## 5 Discussion

In this study we investigated lumbo-pelvic coordination, dynamic stability and movement variability of the trunk in a large sample of participants with low-back pain and asymptomatic controls. We identified sex-specific characteristics in measures of lumbo-pelvic coordination while pain intensity and pain chronicity only had a minor impact. Pain chronicity had an effect on trunk dynamic stability, while variability and dynamic stability of the trunk were not affected by sex and pain intensity.

During the trunk forward bending movement, female participants had a higher lumbar angle (i.e., a higher lumbar lordosis) in the upright standing position due to a higher pelvic angle. The higher pelvic angle in females was maintained throughout the entire movement, resulting in a longer duration of the lumbar lordosis. The fact that the lumbo-pelvic ratio did not differ between females and males during the lordotic phase suggests that the relative position of the pelvis and trunk is maintained in both females and males during this phase. Low-back pain had no effect on pelvic angle or lumbar lordosis, which indicates that the identified lumbo-pelvic posture and kinematics were sex-specific. A similar sex-specific behavior of the lumbo-pelvic coordination during trunk forward bending has been reported for healthy adults (23) and adolescent athletes (56). The lower lumbo-pelvic ratio found in females during the phase of lumbar kyphosis was the reason for a tendency towards a lower lumbo-pelvic ratio in females during the full range of motion (*p* = 0.051). When comparing asymptomatic males and females during a full trunk flexion, females generally displayed a smaller lumbo-pelvic ratio than males (23, 54, 73), mainly due to a greater pelvic range of motion and a greater angle of sacrum orientation in standing and full flexion. Sex-specific differences in sacral shape and orientation (74) or hamstring flexibility (75) might affect the lumbo-pelvic kinematics resulting in a higher pelvic angle in females. It has been argued that in female participants lower muscular strength capacities (76–79) and sex-specific characteristics in muscle morphology (80, 81) may also affect lumbar lordosis and pelvic inclination (26). However, several studies failed to support a relationship between trunk muscle strength, lumbar lordosis and pelvic inclination (56, 82, 83) suggesting that muscle strength may not be the reason for the sex-specific higher lumbar lordosis. The transmission of muscular forces and the resulting stability of the pelvis is dependent on the ligamentous network (84–86) and there is evidence that the pre-tension of the sacrotuberous ligament differ between males and females (87). Hence, sex-specific variations in the pre-tension of the sacrotuberous ligament may have the potential to influence the posture and kinematics of the lumbo-pelvic region (88–90).

A lordotic posture and higher pelvic rotation is associated with an increased loading of the spine (11, 16, 25). Arjmand and Shirazi-Adl (25) reported that lordotic postures increased pelvic rotation and trunk extensor muscle forces with concomitant increases in spine loading. The found effects of sex on lumbar lordosis and pelvic motion may increase trunk loading and can be interpreted as a possible risk factor for a low-back injury in females. Epidemiological studies reporting a higher prevalence of low-back pain in females (91–93) support the higher risk of a low-back injury in females. However, in our results, low-back pain intensity was not associated with lumbo-pelvic posture and kinematics (i.e., no effects of pain on lumbar lordosis, pelvic RoM, duration of lumbar lordosis, lumbo-pelvic ratio), indicating that lumbo-pelvic posture and kinematics may not be suitable measures to differentiate low-back pain patients and may not be a clear risk factor for a low-back injury. Participants with medium to high pain showed a reduced trunk RoM compared to asymptomatic controls, due to a lower trunk angle at the end of the forward flexion, but without differences in lumbar lordosis or pelvic rotation. Similarly, other studies (53, 55) observed no differences in lumbar lordosis and pelvic angle between participants with and without low-back pain. Some previous studies have reported an effect of low-back pain on the lumbo-pelvic ratio, with a relatively greater lumbar contribution during full trunk flexion in participants with low-back pain (19, 22, 94). When classifying patients with low-back pain into specific subgroups, Kim et al. (21) found differences in the lumbo-pelvic ratio between healthy participants and specific subgroups of low-back pain. It has therefore been suggested, that the relative contributions of the lumbar spine and the pelvis to a flexion movement may be of clinical relevance (55) with applications in diagnostics (19, 21, 22, 95), load and injury-risk estimation (13–16) and therapy of low-back pain (17, 18). When pooling data from previous experimental studies in a meta-analytic approach, Laird et al. (55), similar to our study with the large number of participants, found no significant differences in lumbo-pelvic coordination for participants with and without low-back pain. In our study, participants with low to medium pain intensity had a reduced lumbar spine angle when standing upright compared to both asymptomatic controls and participants with medium to high pain intensity. However, the participants with medium to high pain did not differ from the asymptomatic controls, so the results did not show a consistent effect of pain intensity on lumbar spine alignment. We argue that, besides the influence of lumbo-pelvic posture and kinematics on spinal loading (11), it remains unclear whether these variables can influence the prevalence of low-back pain.

We did not detect any significant main effect of sex and pain intensity on trunk dynamic stability and trunk movement variability during the used movement task. There are indications of differences in the variability of trunk movements between people with low-back pain and asymptomatic controls (43, 44). However, the characterization of these modifications is currently not well understood (49). Heterogeneous metrics and inclusion criteria limit the quality of evidence, making it difficult to draw conclusive inferences. Several previous studies also reported no main effect of low-back pain on the spatial variability and local dynamic stability of trunk kinematics during repetitive reaching tasks (39, 96). When reducing pain intensity by implementing an exercise therapy, Arampatzis et al. (50) reported unchanged local dynamic stability of trunk motion despite a significant reduction in low-back pain. Recent reviews, however, show inconsistent results (52) reporting differences (97) or no differences (51) in movement variability between participants with low-back pain and asymptomatic controls. It appears that both the variability and the local dynamic stability of trunk movement may not be sensitive enough to detect an association with low-back pain intensity. Although pain intensity did not affect local dynamic stability, we found higher values of the short-term Lyapunov exponent in chronic low-back-pain patients, indicating higher local trunk instability in these participants. It seems that chronic pain, in our case at least 12 weeks of continuous pain, may induce internal control errors in the regulation of trunk movement during cyclic coordinative tasks involving flexion-extension and rotation, worsening the local dynamic stability of the trunk. These findings indicate that non-linear analysis of trunk movement kinematics may be a useful tool in chronic low-back pain patients and could be considered in the clinical diagnosis and management of low-back-pain.

There are some limitations associated with the approach used in the current study. It is worth noting that with the accelerometers in our study we estimated the sacrum orientation as part of the pelvic girdle and not the pelvic tilt or the curvature of the lumbar spine directly. Still, our results are in agreement with those from previous studies that assessed lumbo-pelvic coordination using noninvasive methods (11, 20, 22, 56, 98) and it has been argued that sacral and pelvic ranges of motion can be assumed to be equal (23). Further, it has been demonstrated that the curvature measured on the back surface significantly correlates with angles measured from x-rays (99) and provides a reasonable accurate measurement of the total lumbar motion (100). To further account for possible differences between sensor position and subdermal anatomical structures, subjects with a BMI >28.0 kg/m^2^ were excluded, yet limiting our results to only a subgroup of the general population. Finally, it can be argued that the age of the participants may influence lumbo-pelvic kinematics. We further analyzed our data regarding participants age and detected an effect of age in measures of lumbo-pelvic kinematics such as the lumbar angle during upright standing (i.e., lumbar lordosis, *p* = 0.039), the lumbo-pelvic ratio (LPR) during the full movement (*p* < 0.001) and the phase of lumbar kyphosis (*p* = 0.006), range of motion of the lumbar spine (*p* < 0.001) and the pelvis during full movement (*p* = 0.004) and during the phase of lumbar lordosis (*p* = 0.032). Importantly, the previously detected main effects of sex, pain intensity and pain chronicity did remain. The findings show, as also reported earlier (23, 101), that age may influence lumbo-pelvic kinematics.

In summary this current study highlights the effects of sex and low-back pain intensity on lumbo-pelvic coordination and trunk kinematics in standing and during trunk flexion. The presented results emphasize sex-specific characteristics in measures of lumbo-pelvic coordination while pain intensity and pain chronicity appear to have a minor impact. Our results suggest that lumbo-pelvic posture and kinematics during a trunk flexion movement have limited practicality in the clinical diagnosis and management of low-back pain. The increased local instability of the trunk during the cyclic coordination task studied indicates control errors in the regulation of trunk movement in participants with chronic low-back pain and could be considered a useful diagnostic tool in chronic low-back pain.

## Data Availability

The datasets presented in this study can be found in online repositories. The names of the repository/repositories and accession number(s) can be found below: https://zenodo.org/records/15044691.
